# Gene Editing in Human Pluripotent Stem Cells: Choosing the Correct Path

**Published:** 2015-11-05

**Authors:** Amar M. Singh, Valeriya V. Adjan Steffey, Tseten Yeshi, Daniel W. Allison

**Affiliations:** 1 Transposagen Biopharmaceuticals, Kentucky, USA; 2 Hera BioLabs, Kentucky, USA

**Keywords:** Pluripotent stem cells, Zinc-finger nucleases, CRISPR/Cas9, TALEN, piggyBac, Gene editing

## Abstract

The recent emergence of targeted nucleases has opened up new opportunities for performing genetic modifications with human pluripotent stem cells (hPSCs). These modifications can range from the creation of a routine knock-out to the more challenging single point-mutation. For both the new and established user, deciding on the best approach for the specific modification of interest can be an arduous task, as new and improved technologies are rapidly and continuously being developed. The choices between the reagents and methodologies depends entirely on the end-goal of the experiments and the locus to be modified. Investigators need to decide on the best nuclease to use for each experiment from among Zinc-Finger Nucleases (ZFNs), Transcription Activator-Like Effector Nucleases (TALENs) and Clustered Regularly Interspaced Short Palindromic Repeats (CRISPR)/Cas9 that would result in the highest likelihood of success with the fewest pitfalls. Furthermore, there have been significant improvements over the first-generation nucleases, such as the development of the dimeric CRISPR RNA-guided Fok1 nucleases (RFNs, marketed as NextGEN™ CRISPR) that reduces the “off-target” mutation rate, providing further options for investigators. Should researchers need to perform a point mutation, then considerations must be made between using single-stranded oligo-deoxynucleotides (ssODN) as the donor for homology-directed repair or utilizing a selection cassette within a donor vector in combination with an excision-only piggyBac™ transposase to leave a seamless edit. In this review, we will provide a general overview of the current technologies, along with methodologies for generating point mutations, while considering both their pros and cons.

## Introduction

Human pluripotent stem cells (hPSCs) have become a preferred cell type for disease-modeling studies and research examining fundamental genetic and developmental biology questions^[1]^. This is largely due to their unlimited proliferative capacity, along with their ability to grow in fully-defined media preventing their differentiation. Moreover, by manipulating the signaling networks that maintain pluripotency^[2]^, hPSCs may be specified to progenitors for each of the three germ layers, the mesoderm, endoderm and ectoderm lineages, and subsequently to a large variety of terminally-differentiated cell-types useful for disease-modeling. Importantly, the use of defined media for self-renewal and differentiation significantly helps to overcome the obstacles associated with heterogeneity, which is common during self-renewal and embryoid body differentiation^[3,4]^, and may increase differentiation efficiencies to >95%.

Over the past decade, site-specific nucleases such as Zinc Finger Nucleases (ZFNs), Transcription Activator-Like Effector Nucleases (TALENs) and Clustered Regularly Interspaced Short Palindromic Repeats (CRISPR)/Cas9 have emerged as a powerful method to perform genetic modifications in human cells^[5]^. Using these site-specific nucleases in hPSCs for performing genetic modifications, whether adding or deleting sequence has become a critical component for disease modelling and basic biological studies. Site-specific nucleases can be used to knock-out a gene by creating an indel (insertion or deletion) or excise genetic elements, such as enhancers altogether^[6,7]^. Another major utility of site-specific nucleases in hPSCs is to create a reporter knock-in into a developmental gene^[8]^, which permits the use of these cells in lineage-tracing experiments that have become commonplace for animal studies. Perhaps the most compelling use for site-specific nucleases in hPSCs is to create point mutations to model genetic diseases^[8]^. This can include creating a mutation that has previously been suggested to correlate with a disease, or to correct a mutation in a patient-derived induced pluripotent stem cell (iPSC).

In this review, we will provide a general overview of the site-specific nucleases and how they function, discussing their known advantages and disadvantages. For further descriptions of these nucleases, more detailed reviews may be examined^[5]^. Finally, we will compare the two most prevalent methods for developing point mutations in hPSCs; that being, the single-stranded oligo-deoxynucleotide (ssODN) method and the seamless selection method with the piggyBac™ transposon system.

## Site-Specific Nucleases

### Zinc-Finger Nucleases (ZFNs)

ZFNs consist of a fusion between the DNA-binding domain of a zinc-finger protein and the nuclease domain of the FokI restriction endonuclease. Two ZFN monomers combine to form a heterodimer that is catalytically active, cleaving DNA to create a double-stranded break^[5]^. A tandem-array of 3-6 zinc-fingers are used to create each monomer that binds 9-18 nucleotide base-pairs, respectively. The half-site for each ZFN monomer is separated by a spacer of 5-7 DNA base-pairs. ZFNs have been used to modify the genomes of numerous cell types in vitro and in vivo, and are currently being examined in clinical studies^[9]^. In general, ZFNs have become less popular than the more recently developed site-specific nucleases (TALENs and CRISPR/Cas9), as there are number of drawbacks to this technology, including its high cost and complexity of building the reagents (Table 1). It should be noted, however, that one advantage of this system over the other nucleases, is that each monomer is much smaller in size than other nucleases, which allows for increased transfection efficiencies and packaging into viruses that can only permit small genomes, such as the adeno-associated virus^[9]^.

The ZFN technology has been demonstrated to work effectively in hPSCs^[10,11]^. This technology has also been used in combination with the piggyBac™ system for seamless editing^[12]^. Specifically, ZFNs were used to bi allelically correct a mutation (E342K) for α1-antitrypsin deficiency in iPSCs. This work was the first example of using site-specific nuclease to correct a mutation in iPSCs, therefore opening the door for disease-modelling and autologous cell-based therapies. Other studies have also utilized ZFN technology to generate a point mutation in PSCs^[13,14]^. However, these studies relied on Cre recombinase to remove the donor vector and upon excision an unwanted loxP site is left behind in the genome, therefore resulting in a stem cell line that has additional changes to the sequence. It should also be noted that the Cre-mediated recombination can have a number of non-specific effects including cytotoxicity and chromosome deletion^[15-17]^, which can preclude this technology from being useful in research and clinical settings.

### Transcription Activator-Like Effector Nucleases (TALENs)

The structure of TALENs are largely similar to ZFNs in that they are composed of heterodimers of a DNA binding domain and the nuclease of the FokI restriction endonuclease. However, instead of using a zinc-finger protein for DNA binding, they use Transcription Activator-Like Effector (TALEs), which were originally identified in plant pathogenic bacteria^[5]^. TALEs consist of tandem repeats of 33-35 amino acids, of which each binds to a single DNA base-pair. Genome editing with TALENs has become more prevalent than the use of ZFNs, as they overcome a number of obstacles such as decreased cytotoxicity and the ability to target nearly any DNA sequence (Table 1). Some studies also indicate that they have reduced off-target mutation rates and mediate higher homology-directed repair compared to other site-specific nucleases, including CRISPR/Cas9^[5,18]^.

There have been a number of studies that have successfully utilized TALEN technology in hPSCs^[8,19]^. Like ZFNs, this technology has also been used in combination with the piggyBac™ system to facilitate seamless removal of the donor cassette^[18,20,21]^. TALENs have also been used in combination with single-stranded oligodeoxynucleotides (ssODNs) to create point mutations in the genome^[22]^. This technology continues to be a popular choice for gene editing in hPSCs, because unlike ZFNs or CRISPR/Cas9, TALENs can bind to nearly any DNA sequence. This therefore provides a clear advantage for nuclease-design strategies.

### Clustered Regularly Interspaced Short Palindromic Repeats (CRISPR)/Cas9

The CRISPR/Cas9 system was originally identified in bacteria as an adaptive immune response to defend itself against bacteriophage. The system was re-appropriated for use as a gene-editing tool only a few years ago, and has had demonstrated success in bacteria, zebrafish embryos and mammalian cells^[5]^. The CRISPR/Cas9 system consists of the Cas9 RNA-guided nuclease along with the crRNA and a tracrRNA, which can be linked together to serve as the guideRNA (gRNA)^[5]^. The largest advantage of the CRISPR/Cas9 system over the TALENs and ZFNs is its ease in design (Table 1). Unlike TALENs and ZFNs which rely on protein-DNA interactions for targeting, CRISPR/ Cas9 relies on gRNA-DNA interactions for targeting. As such, the gRNA sequence can be easily manipulated in vitro to allow for rapid design to target a sequence of interest. The only limitation for gRNA design is that it must bind next to the protospacer adjacent motif (PAM), which is typically a 5’-NGG, which can reduce the number of potential target sites.

Due to its ease of use and lower cost, the CRISPR/Cas9 system is rapidly becoming the prevalent system for genetic manipulation of hPSCs. Numerous studies have demonstrated its efficacy in creating knockouts, deletions and mutations^[6,23,24]^. Several studies have also utilized CRISPR/Cas9 technology with the piggyBac™ transposon system in a seamless manner^[21,25,26]^. Overall, the CRISPR/Cas9 system has proven to be a powerful system for gene editing in hPSCs.

Perhaps the largest drawback of the CRISPR/Cas9 system is the high “off-target” mutation rate. Numerous design algorithms have been developed to circumvent this obstacle, but without full-genome sequencing for numerous clones, the identification of a modified line with little to no non-specific mutations is largely unavoidable^[27]^. This obstacle, however, has been largely overcome by advances in the CRISPR/Cas9 system and specifically the development of dimeric CRISPR RNA-guided FokI nucleases (RFNs, marketed as NextGEN^TM^ CRISPR by Transposagen)^[28,29]^. In this system the dimerization-dependent FokI endonucleases are fused to an inactive Cas9 (dCas9), and two gRNAs are needed facilitate the binding of each dCas9-FokI monomer to their respective half sites. Upon binding of each monomer a catalytically-active dimer forms that creates the double-stranded break. Since two gRNAs are needed for the binding of their respective monomers, this greatly increases the specificity and reduces the “off-target” mutation rate^[28,29]^. Therefore, this system provides all the advantages of the CRISPR/Cas9 and TALEN systems, and none of their drawbacks.

### Designing a Strategy for Generating hPSCs with a Point-Mutation

Two major methods have been utilized for creation of point mutations in hPSCs, the ssODN method and the donor-excision method, and here we will consider their advantages and disadvantages (Table 2). Both of these methods rely on the use of an exogenous donor to facilitate homology-directed repair. The first method uses a single-stranded oligodeoxynucleotide (ssODN) to serve as the donor^[30]^, while the second method relies on the removal of the donor template, following homology-directed repair, either with a Cre recombinase or a piggyBac™ transposase. The piggyBac™ transposase is the preferred method, as this can be used for scarless removal of the donor cassette and therefore results in a truly seamless alteration of the genome. Moreover, as discussed above, Cre recombinase leaves unwanted genetic sequences behind upon donor excision and may have additional detrimental effects on the genome and/or the cells^[15-17]^.

For the first method, an ssODNis built with 60 basepairs flanking each side of the mutation site. To prevent the re-cutting of nuclease, a few base-pair mutations are built into the TALEN or gRNA binding site, such as the PAM sequence. Ideally, these mutations are silent or in intronic regions. It should therefore be noted that since this approach requires sequence changes, and depending on the end-goals of the experiment, this may not be the ideal choice. Following the creation of the ssODN, it is co-transfected with the TALENs or Cas9/gRNA plasmids. Next, the hPSCs may be single-cell cloned or manually isolated by picking individual colonies, and subsequently screened for the mutation of interest. In this method there is no system for selection, so the experimental success is entirely dependent upon the cutting and repair efficiency, which is largely locus-dependent. Typically 1000 or more clones should be screened to identify a correct mutation with no “off-targets”. The major advantage of this system is the rapid timeline. Since there is no lag time needed to create a donor with homology arms, nor any need for selection, a cell line containing the mutation of interest can be developed in as little as 3-4 months.

For the second method, donor-excision, we will specifically consider seamless gene editing using the piggyBac™ system, as this system has clear advantages over Cre recombinase. In this system, a donor plasmid is developed containing 1-2 kb arms for homologous recombination that flank a drug-resistance gene, such as puromycin, followed by thymidine kinase (TK). The drug-resistance gene provides positive selection for homology-directed repair. The thymidine kinase, on the other hand, provides negative selection to isolate those cells in which the donor fragment has been successfully removed following the identification of a modified clone. Typically, the mutation of interest is contained within the donor arms. Also a‘TTAA’ sequence should be present within 200 base-pairs from the mutation site, to allow for excision by the transposase. Once the donor plasmid is prepared, it is transfected along with the nuclease and for CRISPR, the gRNA plasmids. The hPSCs are then selected for approximately 1 week, from which they can then be single-cell cloned or manually isolated, and screened for the mutation of interest. Once an appropriate clone is identified, the donor fragment can be removed using a piggyBac™ transposase with ganciclovir (for TK selection). One problem with this approach can be the potential for random re-integration of the donor following its initial removal. This has been largely overcome by the development of an ‘excision-only piggyBac™ transposase’^[31]^. This transposase is excision-competent, but integration-defective thereby providing the ideal tool for seamless editing. Recent work in iPSCs has confirmed that the excision-only piggyBac™ transposase outperforms both the wildtype and super piggyBac™ transposases for donor removal^[26]^. The only major limitation with this method is that it contains a longer timeline of 4-5 months due the time required for donor creation, along with positive and negative selection. However, the hands-on time required for this approach is generally less as only about 100 clones need to be screened to identify a clone with the modification of interest. Currently this approach is the only system that can be used for developing genetically edited cell lines that are modified in a seamless manner.

## Conclusion

In this review we have provided a general framework of the different site-specific nucleases, along with the advantages and disadvantages, so that investigators can make the best choice for their independent experimental needs. We have described the two most common methods for performing point-mutations in PSCs, both of which have their advantages and disadvantages. From our experience, we find that the most optimal system for generating point-mutations in PSCs is to use either RFNs or TALENs to integrate a selection cassette which can later be seamlessly excised. This provides the highest efficiency, fewest off-target mutations and leaves no unwanted genetic mutations in the modified PSC clone. In sum, the development of site-specific nucleases has opened up many new opportunities for examining disease models and basic biology questions using PSCs. When chosen correctly, the right modification strategies can significantly aide investigators as they pursue their research goals.

## Figures and Tables

**Table 1:** A comparison of site-specific nucleases that can be used for gene editing, range from low (+) to high (+++++).

	ZFNs	TALENs	CRISPR/Cas9	RFNs
**Cost**	+++++	+++	++	++
**Reagent design limitations**	+++	+	++	+++
**Reagent development difficulty**	+++	+++	+	++
**Efficiency**	+++	++++	++++	+++
**Off-target effects**	++	++	+++++	+

Abbreviations: ZFNs, Zinc-Finger Nucleases; TALENs, Transcription Activator-Like Effector Nucleases; CRISPR, Clustered Regularly Interspaced Short Palindromic Repeats; RFNs, dimeric CRISPR RNA-guided *FokI* nucleases.

**Table 2:** A comparison of the methods used to create point-mutations in PSCs.

	ssODNs	Seamless Gene Editing
**Cost**	Moderate	Moderate
**Timeline**	3-4 months	4-5 months
**Reagent design difficulty**	Low	Moderate
**Recommended number of clones for screening**	1000-2000	100-200
**Positive Selection**	No	Yes
**Negative Selection**	No	Yes
**Additional mutations remaining near target site**	Yes	No
**“Hands-on” time**	High	Low
